# Efficacy of an Edible Coating with Carvacrol and Citral in Frozen Strawberries and Blueberries to Control Foodborne Pathogens

**DOI:** 10.3390/foods13193167

**Published:** 2024-10-05

**Authors:** Anna Pié-Amill, Pilar Colás-Medà, Inmaculada Viñas, Irene Falcó, Isabel Alegre

**Affiliations:** 1 Postharvest Biology and Technology Unit, Department of Food Technology, Engineering and Science, University of Lleida, AGROTECNIO-CERCA Center, Av. Rovira Roure 191, 25198 Lleida, Spain; anna.pie@udl.cat (A.P.-A.); pilar.colas@udl.cat (P.C.-M.); inmaculada.vinas@udl.cat (I.V.); 2 Department of Preservation and Food Safety Technologies, Institute of Agrochemistry and Food Technology (IATA), Centro Superior de Investigaciones Científicas (CSIC), Avda. Agustín Escardino 7, 46980 Paterna, Spain; irene.falco@iata.csic.es

**Keywords:** essential oils, fruit, antimicrobial, *Salmonella enterica*, *Escherichia coli* O157:H7, *Listeria monocytogenes*, murine Norovirus

## Abstract

Adding essential oils in an edible coating could be an alternative for the food industry to control foodborne pathogens. In 2014, EFSA published a report highlighting the risk associated with *Salmonella* spp. and Norovirus in fresh and frozen berries. This study aimed to evaluate the efficacy of an edible coating (RP-7) with carvacrol and citral on reducing the population of *Salmonella enterica*, *Escherichia coli* O157:H7, *Listeria monocytogenes*, and murine Norovirus (MNV-1) in frozen strawberries and blueberries. Before evaluating the efficacy, the best method for applying the coating on fruit was studied. The immersion method was selected, with an optimal drying time of 45 min. After this, the berries were frozen and stored for one, two, three, four, and eight weeks at −18 °C. In strawberries, all bacteria were reduced to below 0.7 log cfu/strawberry in the eighth week, and the MNV-1 infectivity showed a reduction of nearly 2 logarithmic units. In blueberries, *S. enterica* and *E. coli* O157:H7 were reduced to 0.8 log cfu/blueberries within a week, and MNV-1 achieved a reduction of 0.8 logarithmic units at the end of the assay. The application of RP-7 affected the studied microorganisms in frozen strawberries and blueberries.

## 1. Introduction

Strawberries (*Fragaria* × *ananassa*) and blueberries (*Vaccinium corymbosum*) are very appreciated fruits by consumers for their texture, taste, and aspect. Their compositions are very rich in vitamins such as vitamin C, lutein, or folates [1] and phenolic compounds like anthocyanins, catechins, and flavonols [2]. Moreover, some authors linked these fruits with preventing and reducing the probability of different diseases. The reason for these effects is attributed to their antioxidant activity, which is related to the reduction of cancer cells, obesity, and inflammation [3,4]. All these nutritional advantages seem to be additional reasons to consume both fruits.

However, the short shelf-life of strawberries is due to their high water content and high breathing rate, which facilitate the proliferation of microorganisms. For this reason, the food industry must apply low temperatures to decrease fruit metabolism and increase their shelf life. Freezing the fruits gives the opportunity to maintain their nutritional and organoleptic properties, minimize oxidative processes, and reduce microbial spoilage [5]. Moreover, consumers can consume them out of season while still retaining the same characteristics as fresh fruit. Frozen fruit is mainly used for smoothie preparation or baking decoration, and thus, it is consumed without any treatment capable of reducing the microbial contamination of the product.

In 2014, the European Food Safety Authority (EFSA) published a scientific opinion highlighting the risk associated with Norovirus and *Salmonella* in fresh and frozen berries [6]. In this opinion, EFSA recommended the implementation of good hygiene practices (GHP), good manufacturing practices (GMP), and good agricultural practices (GAP) for producers to control the risk. Although pathogenic microorganisms were not detected in frozen and fresh strawberry samples taken from different Spanish supermarkets [7] since 2020, 17 notifications of the presence of viruses, bacteria, or molds on berries in the European Union have been made [8].

Therefore, the recommendations of EFSA are not enough to reduce the incidence of foodborne pathogens in berries. Consequently, the food industry needs new strategies to complement the existing ones. Applying physical and chemical methods to the fruit has been the most used approach until now.

Berries must be disinfected before the freezing process. After this step, they are brought into direct contact with liquid nitrogen or carbon dioxide. The contact can be achieved through immersion or spraying (cryofreezing) [9]. The process has a high freezing rate, which minimizes the size of the crystals inside the food, thereby maintaining its fresh characteristics.

Some studies have reported that the application of disinfectants and organic products results in reductions of foodborne pathogens. The addition of peracetic acid (PA) at 80 ppm in the sanitizing water for fresh strawberries reduced *Salmonella enterica* and *Listeria monocytogenes* by more than 2 logarithmic units, and it also showed effectiveness against murine norovirus (MNV-1), with a reduction of 1.9 logarithmic units. In addition, other sanitizers, such as citric acid, lactic acid, and acetic acid, were evaluated [10]. The problem with this type of product is the reduction in efficacy as the organic material in the water increases and its impact on the organoleptic characteristics. On the other hand, ultraviolet light C (UV-C) could be a good option for the food industry. A water-assisted UV-C light prototype combined with PA (80 ppm) reduced the population of *S. enterica* and *L. monocytogenes* by 5 and 7 logarithmic units, respectively, in fresh strawberries [11]. Despite these results, the UV-C light has no residual effect, but it has a reduced penetrability and a high cost [12].

Essential oils (EOs) have been widely used because of their medicinal properties and antibacterial activity [13]. EOs are hydrophobic due to their chemical structure, which may include monoterpenes or sesquiterpenes and be complemented by alcohols, aldehydes, carbohydrates, ethers, ketones, and phenols [14]. This configuration likely contributes to their antimicrobial and antiviral activity. Two of the most studied components of EOs are citral and carvacrol, both of which have been extensively reported for their antibacterial action [15,16]. However, a disadvantage of the EOs is their volatility, so they need a matrix to be retained.

Consuming edible coatings along with the food they wrap or coat is safe, and they can increase the shelf life of the food [17]. These coatings can be used by different methods, such as spraying, immersion, or nano-spraying [18]. They can be made from natural sources such as lipids, proteins, polysaccharides, or combinations, depending on the needs of the product [19]. They can form different types of coatings with distinct properties depending on their composition. Nowadays, the application of edible coatings is growing in the food industry to improve the shelf life of vegetables and fruits, as they act as O_2_, CO_2_, and water vapor barriers [20]. Edible coatings can help protect the composition of the food during its shelf life and reduce weight loss [21]. Applying a coating gives the opportunity to create a sustainable product, which is a green strategy that reduces other types of packaging [22]. Consumers could probably see this innovative technology as an alternative to synthetic additives. Additionally, edible coatings can be formulated with antimicrobial components such as EOs, increasing food safety and product quality. In this case, the antimicrobial effect of EOs can be retained by the coating, which also provides mechanical protection to the fruit.

Food loss is an actual problem for the planet, with almost 14% of food being lost after the harvest [23]. For this reason, the food industry should provide solutions and ensure responsible production. In juice production, certain by-products, such as pulp and other organic materials, are often discarded. Managing these residues adds cost to companies. However, finding alternative uses for these residues could help reduce costs and mitigate food loss. For instance, the pectin in the pulp can be transformed into a powder, which can then be used as a matrix for edible coatings [24].

Our research group has developed the RP-7 edible coating, formulated using pectin to form the edible coating applied to the fruit. Pectin is a biodegradable polymer that is categorized as “Generally Recognized As Safe” (GRAS) [25]. It can be extracted from different sources, mostly fruit peel [26]. The differences in the degrees of methylation depend on their structure. In our study, the pectin had a low degree of methylation, producing a flexible, durable coat permeable to water vapor. These characteristics could be less appreciable than the high methylation degree because they are more rigid and less permeable. The strawberries have a high breath rate; for this reason, the coating has to be permeable. The films created by this polysaccharide can be either continuous crystalline or amorphous coats, depending on its preparation [27]. Two components of EOs, citral and carvacrol, were added to enhance food safety. Citral is a monoterpenoid aldehyde composed of its trans and cis forms [15]. The origin of this component is lemon plants, such as the EO of lemongrass (*Cymbopogon citratus*), where citral constitutes 73% of the oil [28]. Carvacrol is a monoterpene that can be found in the composition of different EOs, such as oregano (*Origanum vulgare*), thyme (*Thymus vulgaris*), and other aromatic plants [29]. In oregano essential oil, carvacrol is the main component, about 85% of the total, with the remainder consisting of other terpenes [30]. Both citral and carvacrol are on the list of “Substances Added to Food” approved by the Food and Drug Administration of the United States (FDA) as flavorings or adjuvants [31].

The aim of this study was to determine the efficacy of an edible coating formulated with pectin and two Eos components (carvacrol and citral) in reducing the population of *S. enterica*, *Escherichia coli* O157:H7, *L. monocytogenes* and MNV-1 (as a norovirus surrogate) artificially inoculated in frozen strawberries and blueberries.

## 2. Materials and Methods

### 2.1. Fruit Samples

Fresh strawberries and blueberries at commercial ripeness were obtained from a local distributor in Lleida, Spain. Fresh fruits were stored at 4 °C until the assays. Just before the strawberry experiment, the peduncle was carefully discarded without causing injury.

### 2.2. Edible Coating (RP-7) Preparation

The edible coating (RP-7) consisted of a citric pectin (Cargill, Wayzata, MN, USA) and two components of EOs, citral (Thermo Fisher, Waltham, MA, USA) and carvacrol (Sigma-Aldrich, St. Louis, MA, USA). The pectin concentration used for the RP-7 coating was 1.5%, and the concentration of EOs components was 0.68%, which is the ratio of carvacrol to citral 1:2.8. For the preparation of the edible coating, the pectin was solubilized in deionized water at 80 °C, mixed with a manual mixer (Quickchef, Moulinex^®^, Alençon, France) at sixth speed for one minute, and stored overnight at 4 °C the day before the experiments. The next morning, the components were added directly to the chilled pectin suspension, and all ingredients were thoroughly mixed using the same manual mixer at the same conditions.

### 2.3. Microorganisms Obtention

#### 2.3.1. Bacterial Strains

The fruits were artificially inoculated with a cocktail of different bacterial strains. Three strains of *S. enterica* subsp. *enterica* were used: ATCC BAA 710 (serovar Montevideo), ATCC BAA 711 (Gaminara), and CECT 4300 (Enteritidis). For *L. monocytogenes*, the three used strains were CECT 940 (serovar 4d), CECT 4031 (serovar ½), and CECT 4032 (serovar 4b). For *E. coli* O157:H7, only one strain was used (ATCC BAA 700728).

All the stains were grown individually at 37 °C for 24 h. The gram-negative bacteria were grown in 100 mL of tryptone soy broth (TSB, Biokar Diagnostics, Beauvis, France), and the gram-positive bacteria were grown in 100 mL of tryptone yeast extract soy broth (TYSEB, TSB supplemented with six g/L of yeast extract (Biokar Diagnostics, Beauvis, France)). After the incubation, the culture of each stain was centrifuged at 9800× *g* for 10 min at 20 °C. Then, all the pellets were resuspended in a sterile saline solution (SS, 8.5 g/L NaCl (PanReac, Barcelona, Spain)). Afterward, different volumes of microorganisms were mixed in a centrifuge tube to achieve 1 × 10^8^ cfu/mL for each strain. The population was checked by plating them into selective media: Xylose Lysine Deoxycholate agar (XLD, Biokar Diagnostics, Beauvis, France) for *S. enterica*, Palcam agar (Palcam, Biokar Diagnostics, Beauvis, France) for *L. monocytogenes*, and MacConkey Sorbitol Agar with Cefixime and Tellurite (CT-SMAC, Biokar Diagnostics, Beauvis, France) for *E. coli* O157:H7.

#### 2.3.2. Viral Propagation, Cell Line and Titration

The viral experiments were done with MNV-1, a surrogate of the human norovirus. For virus propagation and quantification, murine macrophage cell line RAW 264.7 was used. Both were provided by Prof. H. W. Virgin, Washinton University School of Medicine (USA). The RAW 264.7 cells were kept in a humidity incubator (INCO246, Memmert, Schwabach, Germany) at 37 °C with 5% CO_2_ and 95% relative humidity (RH) inside culture flasks of 75 cm^2^ (Nunc, Thermo Fisher, Waltham, MA, USA) with Dulbecco’s modified Eagle medium (DMEM, HyClone, Washington, DC, USA) in a completed form with 10% heat-inactivated fetal serum bovine (FBS, Gibco, Waltham, MA, USA), 1% HEPES (Gibco, Waltham, MA, USA), 1% Penicillin-Streptomycin (Sigma-Aldrich, St. Louis, MA, USA), and 1% L-Glutamine (Gibco, Waltham, MA, USA).

To obtain the MNV-1 stock, a flask of confluent RAW 264.7 cells was infected with MNV-1 without media and incubated for one hour. Then, complete DMEM was added into the flask and returned to the incubator for 40 h. After that, the virus was harvested by three freeze-thaw cycles (−80 °C for one hour and later in the incubator until the ice was melted) of infected cells [32]. Then, all the tissue cultures were transferred to a centrifuge tube centrifuged at 1038× *g* for 20 min, and the supernatant was recovered.

With the aim of quantifying the MNV-1 (stock or samples), RAW 264.7 cells at 80% confluence in a 96-well plate (Nunc, Thermo Fisher, Waltham, MA, USA) were used. That day, the DMEM of cells was removed, and each well was inoculated with serial dilutions of the stock or samples (20 µL/well). After incubation for one hour, 150 µL of DMEM at 2% of FBS was added to each well. The plate was incubated for three days at 37 °C with 5% CO_2_ and 95% RH. After that, monolayers of cells were observed by visual inspection using an optical inverse microscope to see if the cells had cytotoxicity effects, and the 50% tissue culture infectious dose assay (TCID_50_) was applied [33].

### 2.4. RP-7 Application to Fresh Strawberries: Spraying and Immersion

#### 2.4.1. Fruit Inoculation

Each fresh strawberry was previously marked with a square of 1.4 × 1.4 cm (1.96 cm^2^). Then, 50 µL of the bacterial cocktail (1 × 10^6^ cfu/cm^2^) was inoculated in each square. After drying the inoculum, the fruit was stored overnight at 4 °C.

#### 2.4.2. Spraying and Immersion

In this study, two methodologies of RP-7 application were evaluated in fresh strawberries: spraying and immersion.

For spraying, a prototype consisting of a pump, a regulator of the caudal, and spray orifices was used. The coating was applied to the strawberries located in a vertical position to reduce the excess of the coating. For immersion, the fruits were placed in a metallic basket and directly immersed into the RP-7.

After this process, the fruit was dried statically at 4 °C. The drying time was chosen based on the minimum required time: 45 min. Also, 90 and 180 min were tested. Inoculated strawberries without coating were kept under the same conditions as the control treatment. At each sampling time, six RP-7 coated strawberries and six control strawberries (without RP-7 coating) were taken to determine the bacterial population, as described below.

#### 2.4.3. Bacterial Counts in Fresh Strawberries

All the samples were analyzed in triplicate, and each replication was composed of two squares of strawberries. The marked area was cut using a scalpel and put into a blender bag with a filter (BagPage^®^, Interscience, Saint Nom la Bretêche, France). Then, 5 mL of buffered peptone water (BPW, Biokar Diagnostics, Beauvais, France) was added, and the bag was mixed (MiniMix, Interscience, Saint Nom la Bretêche, France) for 90 s at nine strokes/s. The bacterial concentration was determined by plating tenfold dilutions in selective media depending on the microorganisms described previously.

### 2.5. Efficacy of RP-7 on Frozen Fruit

#### 2.5.1. Fruit Inoculation

Fresh strawberries and blueberries were used for this experiment. Strawberries were inoculated superficially with 100 µL of the bacterial cocktail (1 × 10^6^ cfu/fruit) or MNV-1 (1 × 10^6^ pfu/fruit) in small drops. Blueberries were inoculated by immersion for three minutes at 160 rpm in a bath contaminated with 10^8^ cfu/fruit of bacteria or 10^9^ pfu/fruit of MNV-1 [34]. Then, the liquid was dried, and the blueberries were dried inside the laminar flow hood for one hour. Strawberries and blueberries were stored overnight at 4 °C.

#### 2.5.2. RP-7 Application: Immersion and Freezing

Inoculated fruits were immersed in the coating inside a metallic basket. Then, they were dried at 4 °C for 45 min. The control fruits (inoculated strawberries and blueberries without coating) were kept under the same conditions.

After drying, liquid nitrogen was used to freeze both fruits inside an isothermal box for 30 min. The frozen fruits were then introduced in plastic bags and kept at −18 °C for one, two, three, four, and eight weeks. When the stipulated storage times ended, bacterial or viral counts were made directly, without thawing.

#### 2.5.3. Microbial Counts

To determine the bacterial population in strawberries, we placed one frozen strawberry in a blender bag (BlenderBag, Corning, NY, USA) with 5 mL BPW and manually fractionated for 90 s. This process was repeated for three strawberries at each sampling time. Three blueberries were placed in blender bags per replicate. Then, 7 mL of BPW was added, and the bag was agitated for five minutes at 200 rpm. The concentration was determined as described in Section 2.4.3.

The same methodology was used for MNV-1 with one modification: Tris-glycine-beef-extract at 9.5 pH (TGBE, 12.1 g/L Tris base (Scharlau, Barcelona, Spain), 3.8 g/L glycine (Sigma-Aldrich, St. Louis, MA, USA), and 10 g/L beef extract (Sigma-Aldrich, St. Louis, MA, USA) was used as the diluent. The liquid was then transferred to a centrifuge tube and centrifuged at 3000× *g* for ten minutes at 4 °C. The supernatant was stored at −80 °C until the quantifications were performed, as described in Section 2.3.2.

### 2.6. Statistical Data Analysis

The microbiological results were transformed into decimal logarithms (log cfu/cm^2^ or log cfu/fruit). When bacteria could not be counted in the enumeration but were detected after enrichment, the results are expressed as below the detection limit (in this case, below log 0.7 cfu/strawberry and 0.8 log cfu/blueberries). When the pathogen could not be counted and was not detected after enrichment, the results are expressed as non-detection.

All results in this study were analyzed using the JMP Pro 17 statistical software (SAS Institute Inc., Cary, NC, USA). The data obtained were subjected to an analysis of variance (ANOVA) test to determine significant differences between the results. To observe these differences, we applied Tukey’s Honest Significant Difference (HSD) test for means (*p* < 0.05) on the efficacy assays. In all the assays, the Student’s *t*-test was also used to compare treatments at the same time with a significance level of *p* < 0.05.

## 3. Results

### 3.1. RP-7 Application Method: Spraying and Immersion

**Figure 1:**
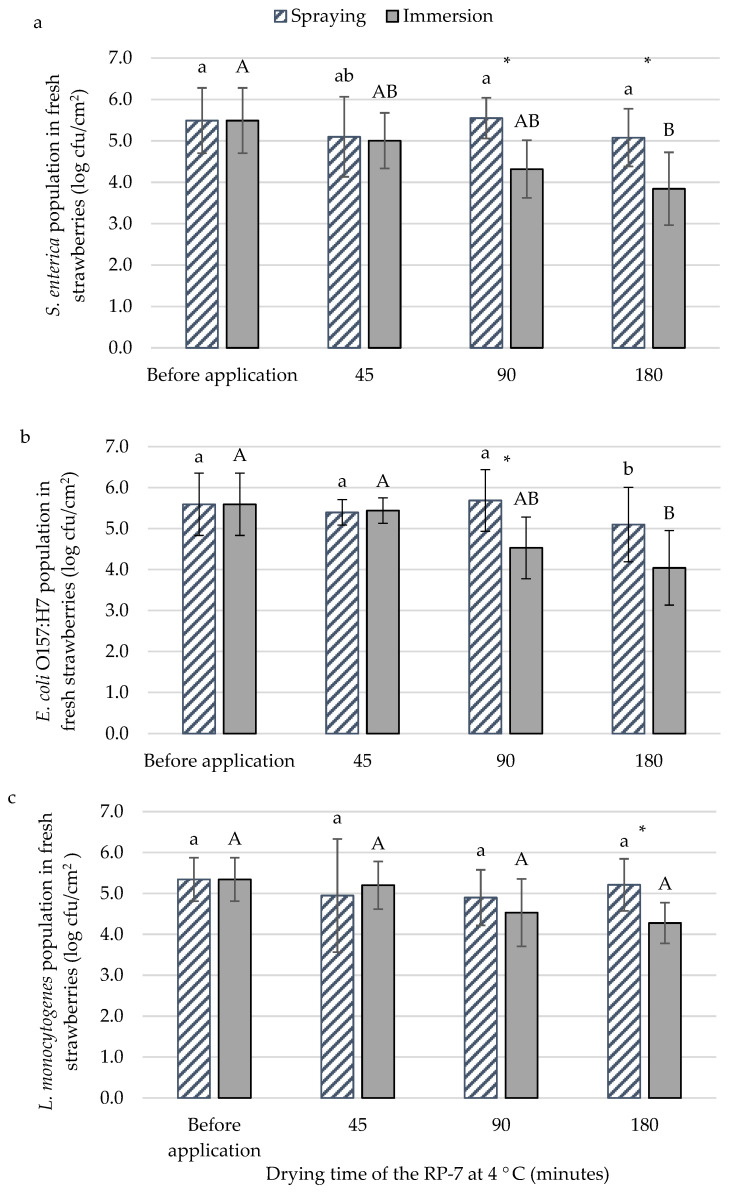
The population of *Salmonella enterica* (**a**), *Escherichia coli* O157:H7 (**b**), and *Listeria monocytogenes* (**c**) with the application of RP-7 with spraying (striped bars) or immersion (solid bars) methods at different drying times of RP-7 on fresh strawberry at 4 °C. Error bars represent the standard deviation of the mean concentrations (n = 6), and the letters indicate the presence of significant differences between drying times at each sampling time according to Tukey’s HSD test (*p* < 0.05). The asterisk (*) indicates the presence of significant differences between treatments at each sampling time according to the Student’s *t*-test (*p* < 0.05).

The best methodology (spraying or immersion) for applying the RP-7 coating against bacteria was evaluated on fresh strawberries. In addition, different drying times (45, 90, and 180 min) in statical refrigeration were studied. The initial populations of *S. enterica* (Figure 1a), *E. coli* O157:H7 (Figure 1b), and *L. monocytogenes* (Figure 1c) were 5.5 ± 0.8 log cfu/cm^2^, 5.6 ± 0.8 log cfu/cm^2^, and 5.3 ± 0.5 log cfu/cm^2^, respectively. For *E. coli* O157:H7 and *L. monocytogenes*, no differences between application methods were observed after 45 min of drying. The *S. enterica* population in immersion application decreased by 1.2 logarithmic units after 90 min of drying compared to spraying. Under the same conditions, *E. coli* O157:H7 reached the same reduction. For *L. monocytogenes*, significant differences compared to the spraying method were not observed until 180 min of drying, achieving a population reduction of nearly one logarithmic unit. In this assay, immersion was the selected method for coating application. Another reason for selecting it was the homogeneity of the layer. The sprayed RP-7 layer was thinner and lacked continuity, resulting in inconsistent application across the samples. The selected time to apply the RP-7 was 45 min since differences between applications for the *E. coli* O157:H7 and *L. monocytogenes* populations were not observed. In fact, for the food industry, it is the best time for the RP-7 application because they need a short time to be profitable.

### 3.2. Efficacy of RP-7 Coating on Frozen Fruit

#### 3.2.1. Effect of RP-7 Coating against Pathogenic Bacteria on Frozen Strawberries and Blueberries

**Figure 2:**
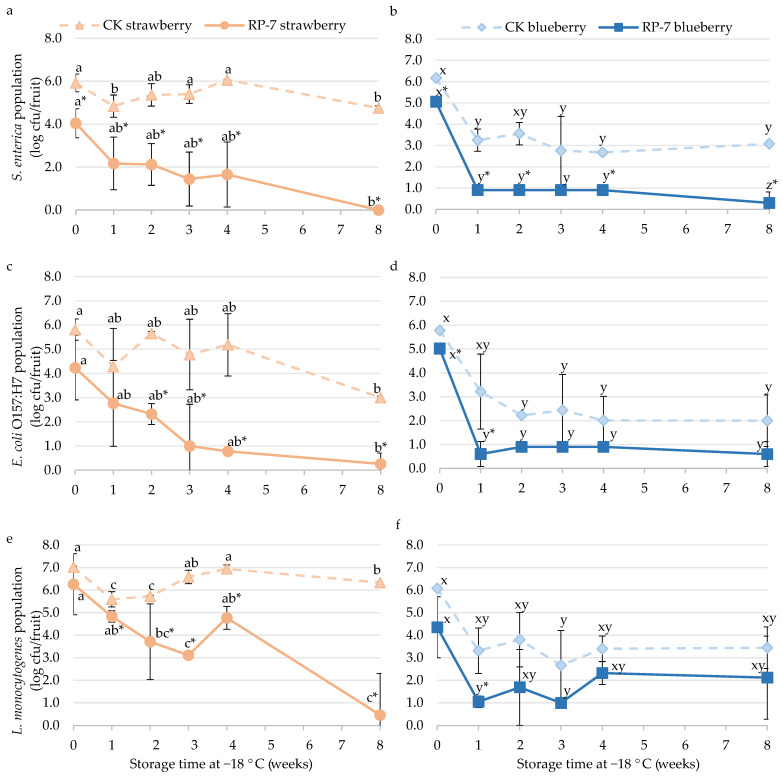
The population of *Salmonella enterica* (**a**,**b**), *Escherichia coli* O157:H7 (**c**,**d**), and *Listeria monocytogenes* (**e**,**f**) on frozen strawberry (**a**,**c**,**e**) and blueberry (**b**,**d**,**f**) coated with RP-7 (solid lines) and without RP-7 (CK, dotted lines) during frozen storage (−18 °C). Error bars represent the standard deviation of the mean concentrations (n = 3), and the letters indicate the presence of significant differences between storage duration in each treatment according to Tukey’s HSD test (*p* < 0.05). The asterisk (*) indicates the presence of significant differences between the two treatments at the same time in each fruit according to the Student’s *t*-test (*p* < 0.05).

After 45 min of drying on strawberries and blueberries, the application of the edible coating RP-7 reduced the population of *S. enterica* by 1.9 and 1.1 logarithmic units (Figure 2a,b), the population of *E. coli* O157:H7 by 1.6 and 0.8 logarithmic units (Figure 2c,d), and the population of *L. monocytogenes* by 0.7 and 1.7 logarithmic units (Figure 2e,f), respectively.

In the case of frozen strawberries, the population of *S. enterica* in the RP-7 coated samples was reduced by almost 3 logarithmic units after the first week at −18 °C (Figure 2a). Over time, this reduction increased, and by the eighth week, the population was below the DL (0.7 cfu/strawberry). In the control samples, the population of *S. enterica* was 4.8 ± 0.1 log cfu/strawberry. For frozen blueberries immersed in RP-7, *S. enterica* needed one week to fall almost below the DL (0.9 ± 0.0 log cfu/blueberries) (Figure 2b). There were also significant differences between the control and the RP-7 samples during the rest of the storage period, with the population reaching 3.1 ± 0.8 log cfu/blueberries in control and below 0.8 log cfu/blueberries in the RP-7 samples at the end of storage.

*E. coli* O157:H7 on frozen strawberries (Figure 2c) showed significant differences between the control and the RP-7 starting in the second week (5.7 ± 0.1 log cfu/strawberry and 2.3 ± 0.4 log cfu/strawberry, respectively), with a reduction of nearly 3 logarithmic units. By the fourth week, the population of the bacteria in the RP-7 samples fell almost below the DL (0.7 log cfu/strawberry), while the control samples maintained at 2.0 ± 1.0 log cfu/strawberry. In frozen blueberries (Figure 2d), the *E. coli* O157:H7 population showed significant differences between treatments at the initial time and continued during the storage time after one week. Initially (time 0), the population in the control was 5.8 ± 0.1 log cfu/blueberries, and the RP-7 was 5.0 ± 0.1 log cfu/blueberries. After one week of storage, the *E. coli* O157:H7 population on coated samples was reduced by more than 2.5 logarithmic units, with the population below the DL (0.8 log cfu/blueberries) till the end of the assay. However, no significant differences were observed between the RP-7 and the control samples at the end of storage.

For *L. monocytogenes* (Figure 2e,f), significant differences in the bacterial population between both frozen fruits with or without RP-7 were observed in the first week. However, in strawberries, it was not until the eighth week that the population in coated samples decreased below the DL (0.7 log cfu/strawberry). In contrast, the bacterial population in the control samples remained at 6.3 ± 0.0 log cfu/fruit. Regarding blueberries, the *L. monocytogenes* population on coated and uncoated fruits decreased by 1.7 and 2.3 logarithmic units, respectively, after one week of frozen storage, showing significant differences between treatments. No differences were observed between treatments at subsequent sampling times.

#### 3.2.2. Effect of RP-7 Coating Against MNV-1 on Frozen Strawberries and Blueberries

**Figure 3:**
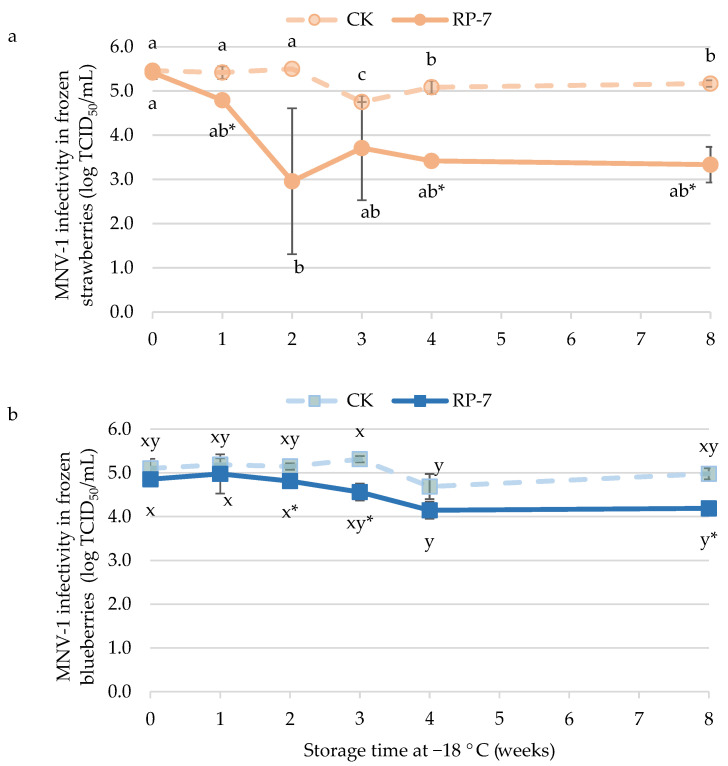
Infectivity of murine norovirus (MNV-1) on frozen strawberries (**a**) and blueberries (**b**) coated with RP-7 (solid lines) and without RP-7 (CK, dotted lines) during frozen storage (−18 °C). Error bars represent the standard deviation of the mean concentrations (n = 3), and the letters indicate the presence of significant differences between storage duration in each treatment according to Tukey’s HSD test (*p* < 0.05). The asterisk (*) indicates the presence of significant differences between the two treatments at the same time in each fruit according to the Student’s *t*-test (*p* < 0.05).

The infectivity of MNV-1 on frozen coated strawberries at the initial time was 5.4 ± 0.1 log TCID_50_/mL (Figure 3a), without significant differences compared to the infectivity on strawberries without the coating. After the first week at −18 °C, the infectivity of MNV-1 in control was maintained at the same level as at the initial time, but in the RP-7 treated samples, MNV infectivity decreased to 4.8 ± 0.1 log TCID_50_/mL. By the end of the storage period, MNV-1 infectivity in the control samples was 5.2 ± 0.1 log TCID_50_/mL, while in RP-7 samples, it was 3.3 ± 0.4 log TCID_50_/mL, which represents a reduction of 1.9 logarithm units.

The efficacy of the RP-7 coating against MNV-1 on blueberries was lower than on strawberries (Figure 3b). The MNV-1 infectivity in the control samples remained stable throughout frozen storage, around 5 log TCID_50_/mL. Significant differences in MNV-1 infectivity between the control and RP-7 samples began in the second week of storage, with a reduction of 0.3 logarithmic units. By the eighth week of storage at −18 °C, a difference of almost 1 logarithmic unit was reached between the control and the RP-7 samples.

## 4. Discussion

This study aimed to demonstrate the efficacy of the RP-7 edible coating on frozen berries against different foodborne pathogens. Experimentation began with the optimization of the application method for RP-7. Immersion was chosen as the technique to apply the RP-7, as it reduced all bacteria populations after 180 min of drying compared to the spraying technique, showing its capability to cover the strawberry better. The spraying method could affect the uniformity of the layer, but this problem could be solved by other spraying techniques, such as electro-spraying or nano-spraying [18]. In the case of immersion, some authors have also applied their coating with the same methodology, dipping the fruit inside their matrix [35,36]. For example, Keynarca et al. [21] observed a reduction in weight loss when the strawberries were immersed in a pectin coating. The second consideration was the contact time needed before freezing the coated inoculated fruits. The selected drying time was 45 min, achieving pathogen reductions of more than 1 logarithmic unit. The selection of the shorter evaluated drying time aligns with the food industry’s needs, where long rest times are incompatible with normal production.

To our knowledge, no research has evaluated the efficacy of an edible coating with components of EOs on frozen berries to control several foodborne pathogens. The action of several EOs was thoroughly studied against different bacteria and viruses [16,37,38]. In this study, the addition of citral and carvacrol to the pectin created a matrix with an antimicrobial effect against the studied microorganisms. The mechanism of action of citral could involve increasing the permeability of the cell membrane and reducing the intracellular pH. These factors could cause a hyperpolarization of the cell membrane [15]. Moreover, other EOs have the same mode of action. For instance, the mustard EO could reduce the internal pH and the intracellular ATP levels of *E. coli* O157:H7 and *Salmonella* Typhi [39]. For *Origanum vulgare* EO, in which carvacrol is one of the main components, Soković et al. [14] reported that a higher antimicrobial effect was reached on gram-positive bacteria than on gram-negative ones. In addition, carvacrol had the highest antimicrobial activity, surpassing even streptomycin. Patterson et al. [37] observed similar results, reporting that oregano oil had more efficacy against gram-positive bacteria. These results could be related to the presence of liposaccharides on the cell membrane of gram-negative bacteria, which stops the diffusion of these EOs. Despite these findings, our study demonstrated that *S. enterica* and *E. coli* O157:H7 were more sensitive than *L. monocytogenes* to RP-7 coating after the first four weeks of storage, regardless of the evaluated fruit. Additionally, Prabuseenivasan et al. [38] did not report differences between gram-positive and gram-negative bacteria applying 21 EOs in an in vitro assay. Certainly, citral and carvacrol have also shown antiviral activity. Gilling et al. [40] observed the mechanism of citral against norovirus; the virus increased its size probably due to the union of the EO with the capsid, therefore inhibiting cell infection.

The application of RP-7 to frozen strawberries reduced *S. enterica*, *E. coli* O157:H7, and *L. monocytogenes* population below the DL (0.7 log cfu/strawberry) for all the microorganisms at the eighth week of frozen storage. In frozen blueberries, the population of *S. enterica* and *E. coli* O157:H7 fell below the DL after one week of storage. However, *L. monocytogenes* was less sensitive than the other bacteria. Other treatments can also be combined with the application of EOs or applied following different methodologies, increasing their efficacy. For example, Liao et al. [41] applied grapefruit EO and UV-C to blueberry, and after one day at 4 °C, the *E. coli* O157:H7 population was reduced by more than 3 logarithmic units. Elsherif et al. [42] applied an EO made by chitosan, rosemary, and carvacrol in an active food packing for pieces of meat, and after seven days at 4 °C, *S. enterica* and *L. monocytogenes* had a reduction in both cases.

The application of RP-7, an edible coating, to frozen strawberries reduced MNV-1 infectivity by nearly 2 logarithmic units by the end of the storage time. In contrast, in blueberries, the reduction was only 0.8 logarithmic units after eight weeks. The results show that MNV-1 is more resistant to the RP-7 than the bacteria in both frozen fruits. The different efficacy observed between fruits could be due to the differences in the surface of the tested fruit. The application of coatings on fresh berries against MNV-1 has been studied by several authors. A coat made of different carrageen applied to blueberries reduced the MNV-1 infectivity less than on raspberries, which fell below the limit of detection after four days at 10 °C [43].

Citral and carvacrol have many properties on human health, such as anticancerogenic, antiviral, antibacterial, antifungal, antibiofilm, or antioxidant activities [44,45]. However, another point to be conscious of is the toxicity of these components of the EOs. Both can be used as a flavor in food products, but there is a lethal median dose (LD_50_). In the case of carvacrol, 810 mg/kg of body weight was reported in rats by oral administration [46]. For citral, LD_50_ on the assays in rats is above 1000 mg/kg of body weight [47]. Nevertheless, some authors highlight the possibility of toxicity in cells. Souza et al. [48] reported that citral has cytotoxicity and genotoxic effects in human cells. For carvacrol, a similar effect can be observed, as it can induce both apoptosis and necrosis in cells [49]. However, in our study, the applied dose was lower than those used in the in vitro assays.

Regardless of the results, physicochemical and sensorial analysis should be performed to know if the application of the evaluated EOs could have an organoleptic impact and changes in the pH, acidity, and soluble solids of fruit. Furthermore, the evaluation of efficacy in other frozen and fresh fruits could be carried out using RP-7.

## 5. Conclusions

The addition of EO components, carvacrol and citral, to a pectin coating improves the retention of the volatile compounds, hence the antimicrobial and antiviral activity. In fact, the immersion method created a uniform layer on the fruit, enhancing effectiveness. This coating provides solutions for the food industry to face the presence of pathogens. However, assays in the pilot plant could give more information about RP-7 to improve the optimization of the application.

To sum up, the application of RP-7 to frozen strawberries and blueberries could be a good strategy to control *S. enterica*, *E. coli* O157:H7, *L. monocytogenes*, and MNV-1 in these frozen fruits.

## Data Availability

The data presented in this study are available on request from the corresponding author.
